# Dual time-point ^18^F-FDG PET/CT imaging with multiple metabolic parameters in the differential diagnosis of malignancy-suspected bone/joint lesions

**DOI:** 10.18632/oncotarget.17140

**Published:** 2017-04-17

**Authors:** Chen-Tian Shen, Zhong-Ling Qiu, Zhen-Kui Sun, Wei-Jun Wei, Hong-Jun Song, Xin-Yun Zhang, Quan-Yong Luo

**Affiliations:** ^1^ Department of Nuclear Medicine, Shanghai Jiao Tong University Affiliated Sixth People’s Hospital, Shanghai 200233, People’s Republic of China

**Keywords:** dual time-point imaging, ^18^F-FDG PET/CT, metabolic tumor volume, total lesional flycolysis, bone/joint lesions

## Abstract

The purpose of this study was to evaluate the diagnostic potential of dual time-point^18^F-FDG PET/CT imaging with multiple metabolic parameters in malignancy-suspected bone/joint lesions. Fifty seven consecutive patients were recruited. PET parameters including SUVmax, SUVmean, metabolic tumor volume (MTV), total lesional glycolysis (TLG) and retention indexes (RIs) were obtained. Thirty five malignant and 22 benign lesions were confirmed by pathology. In all, 48 receiver operating characteristic (ROC) curves were derived. For SUVmax, MTV2.0, TLG2.0, MTV2.5 and TLG2.5, areas under the curves (AUCs) of early time-point imaging were similar to those of delayed time (P > 0.05), while higher than those of dual time (P< 0.05). For MTV50%max, TLG50%max, MTV75%max and TLG75%max, AUCs of early time-point imaging were lower than those of delayed time (P< 0.05), while similar to those of dual time (P> 0.05). In conclusion, dual time-point^18^F-FDG PET/CT imaging shows limited value in the differential diagnosis of malignancy-suspected bone/joint lesions. However, MTV and TLG at a fixed SUV threshold (50% or 75% of SUVmax) in delayed time-point imaging may provide better diagnostic accuracy

## INTRODUCTION

Skeletal sarcoma is a relatively rare and hetero-geneous tumor group. Although most common causes of cancer death are cancers of the lung and bronchus, prostate, breast and colorectum, primary malignancy of the bone/joint is ranked as the third leading cause of death in patients with cancer who are younger than 20 years [1]. By providing important information like the appearance, intraosseous extent and internal characteristics of intraosseous lesions, radiographs (including X-ray and computer tomography [CT]) and magnetic resonance imaging (MRI) are of great importance in the clinical evaluation of skeletal diseases [2]. Meanwhile, ^18^F-fluorodeoxyglucose positron emission tomography/computer tomography (^18^F-FDG PET/CT) is now increasingly used as a powerful evaluation modality in clinical oncology based on its unique ability to detect glycolytic metabolism in tumor cells combined with accurately anatomic location [3]. And it has been used to differentiate malignant and benign diseases including skeletal lesions [4].

^18^F-FDG is not a tumor specific imaging probe. Although the uptake of FDG by malignant tumor cells is generally higher than by benign ones, quantities of exceptions do exist in ^18^F-FDG PET/CT imaging. Multiple studies have shown that dual time-point imaging (DTPI) of ^18^F-FDG PET/CT could enhance the diagnostic accuracy and retention index (RI) of the maximum standardized uptake value (SUVmax) is the most commonly used PET metabolic parameter in DTPI [5–7]. However, few series reported the quantitative analysis of dual time-point and/or delayed PET imaging in differentiating malignant bone lesions from benign ones [8, 9]. Recently, novel quantitative PET parameters including the metabolic tumor volume (MTV) and total lesional glycolysis (TLG) have been proposed [10–12]. These measurements are able to provide volumetric information on glucose metabolism of the tumor. Most studies focus on their prognostic values while their diagnostic potentials in clinical oncology remain undetermined.

To the best of our knowledge, no study has investigated these two different approaches (kinetic parameters like RI and new quantitative PET parameters like MTV and TLG) in a same cohort of patients with the purpose of differentiating malignant lesions from benign processes. Hence, in the current study, we evaluated the diagnostic potentials of dual time-point ^18^F-FDG PET/CT imaging with parameters including SUVmax, SUVmean, MTV, TLG in malignancy-suspected bone/joint lesions.

## RESULTS

### Patients’ characteristics

**Figure 1:**
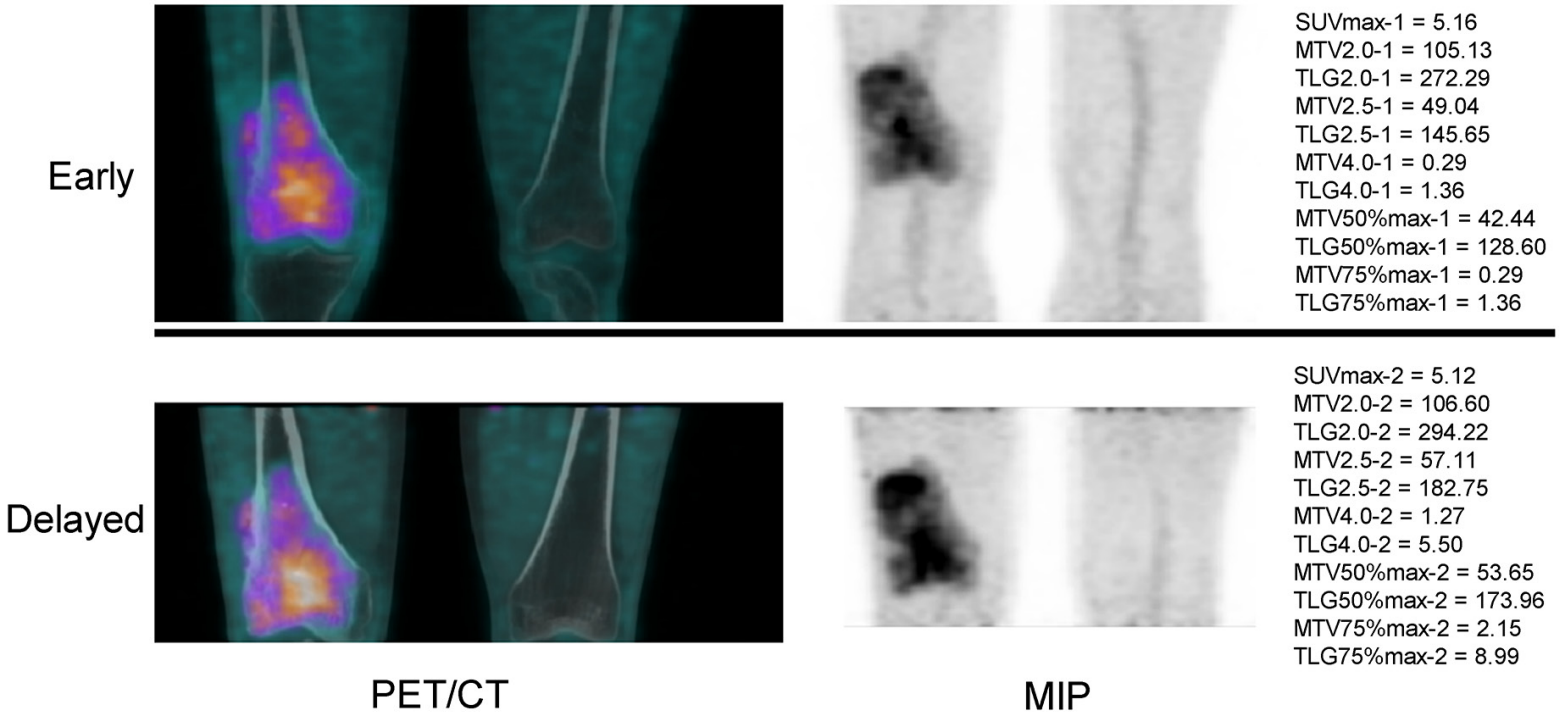
^18^F-FDG PET/CT imaging of a 47-year-old male who was diagnosed of osteosarcoma after surgery MIP, maximal intensity projection.

Fifty seven malignancy-suspected bone/joint lesions in fifty seven individual patients (median age 55 years, range 7-85 years; male/female, 29/28) were evaluated in the current study. Results of histology/cytology revealed 35 malignant and 22 benign lesions. According to final diagnosis, patients were divided into two groups, 35 malignant lesions in malignant group (M) and 22 benign lesions in benign group (B), for further analysis. Chondrosarcoma (17.14%) and osteosarcoma (17.14%) were the most frequently diagnoses in group M. The top three locations of malignant and benign lesions were found in femur (28.57%), pelvis (28.57%), vertebrae (20.00%) and rib (22.73%), pelvis (18.18%), vertebrae (18.18%), respectively. Characteristics including age and gender showed no significant differences between group M and group B. Table 1 demonstrates the basic characteristics of all included patients. Figure 1 shows dual time-point ^18^F-FDG PET/CT imaging of a 47-year-old male who was diagnosed of osteosarcoma after surgery.

**Table 1:** Basic characteristics of included patients, results of pathology (histology/cytology) and locations of lesions

	Malignant lesions	Benign lesions	P
Patient number	35	22	
Age(median/range)	54/7-85	56.5/39-73	0.17
Gender M/F	21/14	8/14	0.14
Diagnosis/n			
	Chondrosarcoma/6	Inflammation/12	
	Osteosarcoma/6	Fracture/2	
	Metastasis/4	Osteofibrous dysplasia/2	
	Multiple myeloma/3	Hemangioendothelioma/1	
	Plasmacytoma/3	Eosinophilic granuloma/1	
	Ewing’s sarcoma/2	Giant cell tumor of bone/1	
	Liposarcoma/2	Osteoarthritis/1	
	Undifferentiated high-grade pleomorphic sarcoma /2	Osteochondritis/1	
	Aggressive giant cell tumor of bone/1	Osteomyelitis/1	
	Chordoma/1		
	Leiomyosarcoma/1		
	Spindle cells malignant tumor/1		
	Squamous cell carcinoma/1		
	Synoviosarcoma/1		
	Undifferentiated sarcoma/1		
Location/n			
	Femur/10	Rib/5	
	Pelvis/10	Pelvis/4	
	Vertebrae/7	Vertebrae/4	
	Humerus/3	Clavicle/3	
	Tibia/2	Femur/2	
	Phalanx/1	Tibia/2	
	Radius/1	Humerus/1	
	Rib/1	Sternum/1	

### PET parameters

All data are shown as median/25-75 percentile. Metabolic parameters of all lesions including SUVmax, MTV2.0, SUVmean2.0, TLG2.0, MTV2.5, SUVmean2.5, TLG2.5, MTV4.0, SUVmean4.0, TLG4.0, MTV50%max, SUVmean50%max, TLG50%max, MTV75%max, SUVmean75%max, and TLG75%max were obtained or calculated accordingly in early time-point and delayed time-point imaging. For dual time-point imaging analysis, RI of these parameters were calculated and compared between group M and group B.

### Early – time point

In early time-point imaging, the values of SUVmax, MTV2.0, SUVmean2.5, SUVmean4.0 and SUVmean75%max were found to have significant difference between group M and group B (SUVmax-1, 6.2/4.20-8.62 vs. 4.22/3.15-6.39, P=0.02; MTV2.0-1, 45.47/21.57-109.17 vs. 6.16/3.42-21.32, P=0.02; SUVmean2.5-1, 3.68/2.98-4.26 vs. 3.09/2.80-3.64, P=0.02; SUVmean4.0-1, 4.97/4.18-5.46 vs. 2.15/0.00-5.12, P=0.03; SUVmean75%-1, 5.45/3.59-7.17 vs. 3.74/2.70-5.81, P=0.04) (Supplementary Table 1). Other parameters showed no significant differences between the two groups in early time-point imaging.

### Delayed – time point

In delayed time-point imaging, the values of SUVmean2.0, MTV2.0, TLG2.0 and SUVmean2.5 were found to have significant difference between group M and group B (SUVmean2.0-2, 3.44/2.73-4.39 vs. 2.79/2.40-3.23, P=0.02; MTV2.0-2, 47.62/22.52-117.67 vs. 7.48/1.96-24.16, P=0.01; TLG2.0-2, 179.29/72.05-494.84 vs. 22.72/5.43-68.41, P=0.04; SUVmean2.5-2, 4.07/3.09-4.90 vs. 3.24/2.91-3.70, P=0.01) (Supplementary Table 1). Other parameters showed no significant differences between the two groups in delayed time-point imaging

### Dual – time point

For dual time-point imaging analysis, retention indexes of SUVmean2.0, MTV50%max, TLG50%max, MTV75%max, and TLG75%max were found to have significant difference between group M and group B (RI-SUVmean2.0, 0.09/0.05-0.13 vs. 0.05/0.02-0.08, P=0.04; RI-MTV50%max, -0.02/-0.20-0.07 vs. -0.24/-0.34--0.044, P=0.02; RI-TLG50%max, 0.10/-0.02-0.25 vs. -0.10/-0.24--0.024, P=0.01; RI-MTV75%max, 0.10/-0.02-0.25 vs. -0.32/-0.57--0.17, P=0.002; RI-TLG75%max, 0.33/-0.10-0.76 vs. -0.21/-0.45--0.13, P=0.0005) (Supplementary Table 1). Other parameters showed no significant differences between the two groups in dual time-point imaging analysis

### ROC analysis

ROC curves of 16 metabolic parameters for their early, delayed and dual time-point imaging were derived with respective AUC, sensitivity, specificity, PLR and NLR and their 95 % confidence intervals (Supplementary Table 2). In all, 48 ROC curves were derived. For each parameter, ROC curves of early (1), delayed (2) and dual time-point (RI) imaging were compared (Table 2)

**Table 2:** AUCs comparisons of early (1), delayed (2) and dual time-point (RI) imaging in different metabolic parameters

	Imaging	AUC	P
SUVmax	1 vs. 2	0.684 vs. 0.659	0.27
	1 vs. RI	0.684 vs. 0.484	**0.03**
	2 vs. RI	0.659 vs. 0.484	**0.04**
MTV2.0	1 vs. 2	0.818 vs. 0.795	0.72
	1 vs. RI	0.818 vs. 0.574	**0.02**
	2 vs. RI	0.795 vs. 0.574	**0.03**
Mean2.0	1 vs. 2	0.664 vs. 0.681	0.54
	1 vs. RI	0.664 vs. 0.687	0.80
	2 vs. RI	0.681 vs. 0.687	0.93
GLT2.0	1 vs. 2	0.814 vs. 0.805	0.43
	1 vs. RI	0.814 vs. 0.603	**0.00**
	2 vs. RI	0.805 vs. 0.603	**0.02**
MTV2.5	1 vs. 2	0.736 vs. 0.768	0.45
	1 vs. RI	0.736 vs. 0.527	**0.04**
	2 vs. RI	0.768 vs. 0.527	**0.03**
Mean2.5	1 vs. 2	0.663 vs. 0.688	0.21
	1 vs. RI	0.663 vs. 0.541	0.50
	2 vs. RI	0.688 vs. 0.541	0.94
GLT2.5	1 vs. 2	0.741 vs. 0.763	0.34
	1 vs. RI	0.741 vs. 0.544	**0.02**
	2 vs. RI	0.763 vs. 0.544	**0.02**
MTV4.0	1 vs. 2	0.663 vs. 0.671	0.90
	1 vs. RI	0.663 vs. 0.579	0.30
	2 vs. RI	0.671 vs. 0.579	0.34
Mean4.0	1 vs. 2	0.660 vs. 0.656	0.38
	1 vs. RI	0.660 vs. 0.643	0.48
	2 vs. RI	0.656 vs. 0.643	0.71
GLT4.0	1 vs. 2	0.657 vs. 0.661	0.68
	1 vs. RI	0.657 vs. 0.556	0.39
	2 vs. RI	0.661 vs. 0.556	0.39
MTV50%max	1 vs. 2	0.659 vs. 0.712	**0.02**
	1 vs. RI	0.659 vs. 0.686	0.83
	2 vs. RI	0.712 vs. 0.686	0.83
Mean50%max	1 vs. 2	0.686 vs. 0.662	0.29
	1 vs. RI	0.686 vs. 0.562	0.20
	2 vs. RI	0.662 vs. 0.562	0.26
GLT50%max	1 vs. 2	0.716 vs. 0.781	**0.02**
	1 vs. RI	0.716 vs. 0.732	0.77
	2 vs. RI	0.781 vs. 0.732	0.78
MTV75%max	1 vs. 2	0.630 vs. 0.790	**0.04**
	1 vs. RI	0.630 vs. 0.769	0.25
	2 vs. RI	0.790 vs. 0.769	0.70
Mean75%max	1 vs. 2	0.675 vs. 0.643	0.17
	1 vs. RI	0.675 vs. 0.531	0.11
	2 vs. RI	0.643 vs. 0.531	0.19
GLT75%max	1 vs. 2	0.705 vs. 0.819	**0.01**
	1 vs. RI	0.705 vs. 0.794	0.09
	2 vs. RI	0.819 vs. 0.794	0.77

Abbreviations SUV, standardized uptake value; Mean, SUVmean; MTV, metabolic tumor volume; TLG, total lesional glycolysis; RI, retention index; AUC, areas under the curve.

**Figure 2:**
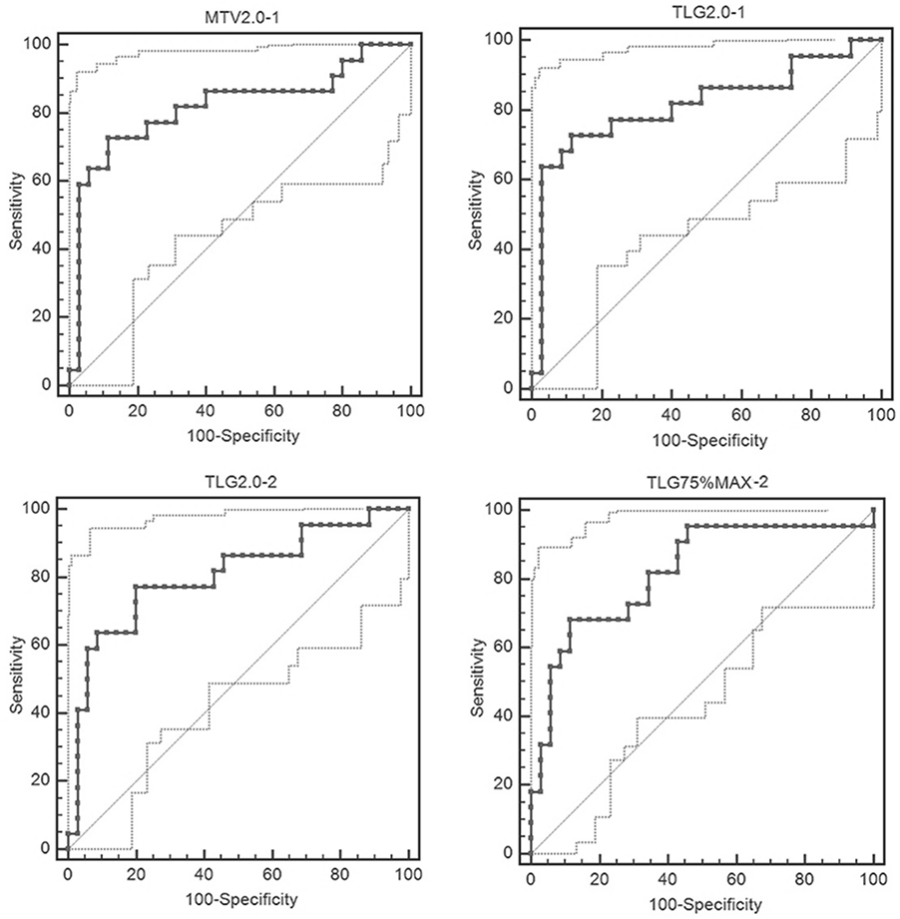
ROCs of MTV2.0, TLG2.0 in their early time-point imaging and TLG2.0, TLG75%max in their delayed time-point imaging with the AUCs more than 0.800

For SUVmax, MTV2.0, TLG2.0, MTV2.5 and TLG2.5, AUCs of early time-point imaging were similar to those of delayed time (P > 0.05), while higher than those of dual time (P< 0.05). For MTV50%max, TLG50%max, MTV75%max and TLG75%max, AUCs of early time-point imaging were smaller than those of delayed time (P< 0.05), while similar to those of dual time (P > 0.05). Figure 2 demonstrates ROCs of MTV2.0, TLG2.0 in their early time-point imaging and TLG2.0, TLG75%max in their delayed time-point imaging, whose AUCs were more than 0.800.

## DISCUSSION

In the current study, diagnostic performance of early, delayed and dual time-point ^18^F-FDG PET/CT imaging in 57 consecutive patients with malignancy-suspected bone/joint lesions were evaluated and compared by using FDG metabolic parameters (SUVmax, SUVmean, MTV, TLG) derived ROC curves. In all, 48 ROC curves from 16 parameters in respective early, delayed and dual time-point imaging were analyzed with their respective AUC, sensitivity, specificity, PLR and NLR. The results demonstrated that for all of the 16 metabolic parameters, diagnosis accuracy of dual time-point imaging showed no superior to that of single (early and delayed) time-point imaging. For SUVmax, MTV2.0, TLG2.0, MTV2.5 and TLG2.5, diagnosis accuracy of dual time-point imaging was much lower than that of early time-point imaging. For MTV50%max, TLG50%max, MTV75%max and TLG75%max, diagnosis accuracy of delayed time-point imaging was better than that of early time-point imaging.

Imaging performed with both standard and prolonged tracer uptake periods is termed “dual time-point imaging,” and dual time-point ^18^F-FDG PET imaging has been reported for almost twenty years [13, 14]. Lots of studies have shown that its diagnostic accuracy is higher when compared to standard imaging (early point-time) by efficiently differentiating malignancy from benign processes [14–19]. However, the clinical utility of ^18^F-FDG PET DTPI remains controversial because the results of a number of other studies have demonstrated marked overlap of FDG uptake patterns between malignant and benign lesions on dual time-point images [5, 20–24]. The underlying rationale to use DTPI to distinguish benign and malignant diseases is that FDG uptake and clearance depend on the time interval between intravenous FDG administration and imaging [25]. Differences of expression of glucose handling key enzymes like glucose-6-phosphatase [26] and glucose transporter-1 [27], metabolic rate and proliferation rate in tumor and non-tumors cells could contribute to the differentiating diagnosis using ^18^F-FDG PET DTPI. Additionally, blood pool and urinary tract clearance of FDG could be approved to lower background activity with a longer distribution time [25, 28]. In addition, because SUV reflects the glucose transporter activity and RI reflects the hexokinase activity, the combination of these two parameters may provide better diagnostic potential.

Although multiple kinds of tumors have been evaluated, few series reported the quantitative analysis of dual time-point and/or delayed PET imaging in differentiating malignant bone lesions from benign ones. Tian et al. [9] compared the SUVmax of primary bone tumors in DTPI in the context of differentiating malignant from benign bone lesions. The AUC for the SUVmax in early time-point imaging (SUVmaxE) and RI-SUVmax in dual time-point imaging were 0.597 (95%CI 0.511–0.707) and 0.757 (95%CI 0.622–0.816), respectively. The AUCs for RI were statistically higher than those for the SUVmaxE (P=0.03). However, this could not be substantiated by our results. We found that the AUCs for the SUVmax in early time-point imaging (SUVmax-1) and RI-SUVmax in dual time-point imaging were 0.684 (95%CI 0.548-0.801) and 0.484 (95%CI 0.350-0.621), respectively and the AUC for RI were statistically lower than that for the SUVmax-1 (P=0.04). Many factors could contribute to this difference. The most important one was the fact that the value of SUVmax in 89.47% (51/57) of patients from our study increased in delayed time-point imaging.

The strengths of our current research were that it included consecutive patients with a relative long follow-up time; it was the first study trying to differentiate malignances from benign processes by combining dual time-point imaging and new PET metabolic parameters (MTV and TLG) and all lesions analyzed were confirmed by pathology (histology/cytology). Abgral et al. [29] first investigated these two different approaches in a cohort of patients for prognostic purpose. They tried to identify the potential correlation of percentage variation of metabolic tumor burden calculated by dual-time point ^18^FDG PET/CT imaging with patients’ recurrence-free survival, and to investigate the prognostic interest of RI and MTV, TLG in comparison with SUVmax in patients with head and neck squamous cell carcinoma (HNSCC). Their results did not prove a prognostic interest of percentage variation of metabolic tumor burden in HNSCC patients. The results of our current study demonstrated that MTV and TLG (MTV50%max, TLG50%max, MTV75%max and TLG75%max) had better diagnostic potential in delayed time-point imaging than early time-point imaging.

There are some limitations in our study. Firstly, the small sample size and various kinds of bone tumors included made it not possible to determine the prognostic value of PET parameters with early/delayed dual time-point imaging. Also, because of the small sample size and a wide range of histologic findings, future study with a large sample size and specific tumor type is wanted to further verify the results. Secondly, it was performed in a single institution, which restricts generalizing the results. Thirdly, diagnosis accuracy between PET alone and PET/CT was not compared, because in the current study CT was used for attenuation correction and lesion location while not for diagnosis purpose. Finally, the second acquisition (PET-2) was performed at 2 hours after ^18^F-FDG injection, which could be argued that the time interval was not long enough to generate a good result. However, although 3 hours was recommended [25], the optimal time interval between the radiotracer injection and delayed time-point imaging is still undetermined.

In conclusion, dual time-point ^18^F-FDG PET/CT imaging showed limited value in the differential diagnosis of malignancy-suspected bone/joint lesions. However, MTV and TLG at a fixed SUV threshold of 50% or 75% of SUVmax in delayed time-point imaging may provide better diagnostic accuracy.

## MATERIALS AND METHODS

The current study was performed at a single institution from January 2011 to December 2012 in Shanghai Jiao Tong University Affiliated Sixth People’s Hospital. This study was approved by the ethics committee of our institution and written informed consent was obtained from all patients (for patient whose age was younger than 18 years, written informed consent was obtained from his/her statutory guardian additionally). And all methods were performed in accordance with the relevant guidelines and regulations.

### Study population

In all, 57 consecutive patients (median age 55 years, range 7-85 years; 29 males, 28 females) were recruited. Patients with malignancy-suspected bone/joint lesions found by X-ray, CT or MRI were invited to participate when they were referred for ^18^F-FDG PET/CT. And a planned biopsy or surgery would be performed in our institution in 2 weeks after ^18^F-FDG PET/CT scan.

Exclusion criteria were a history of received treatments like chemotherapy, radiotherapy or targeted therapy, whether currently/suspected pregnant or breast-feeding, or considered unable to cooperate.

### Imaging technique

Patients were required to fast for at least 6 hours before the ^18^F-FDG PET/CT scan. The blood glucose level was determined before tracer injection, and a maximum value of 11 mmol/L was allowed. The tracer ^18^F-FDG was administered intravenously in a dose of 3.7 MBq/Kg. PET/CT scanning was performed using GE Discovery VCT (General Electric Medical Systems, Milwaukee, WI, USA) with the following settings: CT scan, 120 kV and 80 mA, 64 slices, a slice thickness of 3.75 mm. PET scans were performed in 3D, with a scan time of 2.5 min/bed. Images were reconstructed iteratively by using ordered subset expectation maximization (OSEM). Attenuation correction was used by CT.

All the patients underwent dual time-point imaging. The first acquisition (PET-1) was performed after 60 minutes (± 10 minutes) of ^18^F-FDG injection from the skull to the proximal femur (for patient whose suspected lesion located in the lower limbs, skull to toes was scanned), applying a CT scan, and was immediately followed by a PET scan of the same area. The second acquisition (PET-2) was performed after 120 minutes (± 10 minutes) and was carried out in the position of the suspected lesion found in X-ray, CT or MRI only.

### FDG PET image interpretation and metabolic parameter measurement

Interpretation of the images and data analysis were performed independently by two nuclear medicine physicians who were aware of the patients’ clinical history, which was provided by the referring physician, but were blinded to the potential diagnosis of other imaging studies. Disagreements were further evaluated and resolved by a third physician. Average values of PET parameters were used for further analysis.

A volume of interest (VOI) was placed over the identified bone lesion. PET parameters including SUVmax, SUVmean2.0(fixed SUV threshold of 2.0), SUVmean2.5(fixed SUV threshold of 2.5), SUVmean4.0(fixed SUV threshold of 4.0), SUVmean50%max(fixed SUV threshold of 50% of SUVmax), SUVmean75%max(fixed SUV threshold of 75% of SUVmax), MTV2.0 (fixed SUV threshold of 2.0), MTV2.5(fixed SUV threshold of 2.5), MTV4.0(fixed SUV threshold of 4.0), MTV50%max(fixed SUV threshold of 50% of SUVmax) and MTV75%max(fixed SUV threshold of 75% of SUVmax) were measured for both time-points: early time-point (parameter-1) and delayed time-point (parameter-2). The TLG value (TLG2.0, TLG2.5, TLG4.0, TLG50%max and TLG75%max) was calculated according to the formula: TLG = SUVmean x MTV (with corresponding SUV threshold). Retention indexes (RI) between all of these dual time-point parameters were calculated according to the formula:

RI-X=(X-2 − X-1)/X-1; X=SUVmax, SUVmean2.0, SUVmean2.5, SUVmean4.0, SUVmean50%max, SUVmean75%max, MTV2.0, MTV2.5, MTV4.0, MTV50%max, MTV75%max, TLG2.0, TLG2.5, TLG4.0, TLG50%max or TLG75%max.

### Reference standard

All included patients underwent biopsy or surgery in 2 weeks after ^18^F-FDG PET/CT scan. Histological/cytological results were served as standard of references and all lesions evaluated were proved by pathology in the current study. Twenty nine patients (50.88%) were finally diagnosed through CT-guided percutaneous biopsy, while 28 patients (49.12%) were confirmed by pathology after lesions resection. In clinical practice, it is well known that biopsy could be false negative due to the inexact location of the specimen achieved. To decrease/avoid this possibility, repeated biopsies were performed in 3 patients. In addition, we followed up all these patients for a relative long time (51.49±5.04 months) to further insure the accuracy of the final diagnosis.

### Statistical analysis

Receiver operating characteristic (ROC) curves and respective areas under the curve (AUC), sensitivity, specificity, positive likelihood ratio (PLR) and negative likelihood radio (NLR) with their 95 % confidence intervals (95 % CIs) were determined by using MedCalc® version 11.4.2.0, 64-bit (MedCalc Software bvba, Ostend, Belgium). ROC curves were compared based on the nonparametric approach proposed by Hanley & McNeil, 1982 [30]. Comparisons of continuous variables between two groups were performed using Student’s t test (assuming equal variances) or Welch-test (assuming unequal variances) while comparisons of categorical variables were performed by using the chi-square statistic. Statistical significance was assumed at a P value of less than 0.05.

## SUPPLEMENTARY MATERIALS TABLES







## Abbreviations

CT: computer tomography
MRI: magnetic resonance imaging
^18^F-FDG PET/CT: ^18^F-fluorodeoxyglucose positron emission tomography/computer tomography
DTPI: dual time-point imaging
RI: retention index
SUVmax: maximum standardized uptake value
MTV: metabolic tumor volume
TLG: total lesional glycolysis
AUC: areas under the curve
PLR: positive likelihood ratio
NLR: negative likelihood radio
CI: confidence intervals
