# Supplementary material for: Dual time-point 18F-FDG PET/CT imaging with multiple metabolic parameters in the differential diagnosis of malignancy-suspected bone/joint lesions
Source: Oncotarget. 2017 Apr 17;8(41):71188–96. doi: 10.18632/oncotarget.17140 (PMC5642630; doi:10.18632/oncotarget.17140)
Supplement: Supplementary file 1 [file oncotarget-08-71188-s001.pdf]

Dual time-point ^{18}F-FDG PET/CT imaging with multiple metabolic parameters in the differential diagnosis of malignancy-suspected bone/joint lesions

SUPPLEMENTARY MATERIALS

SUPPLEMENTARY TABLES

Supplementary Table 1: Values of different metabolic parameters in their early, delayed and dual time-point imaging.

See Supplementary File 1

Supplementary Table 2: AUC, sensitivity, specificity, PLR and NLR and their 95% confidence intervals of different metabolic parameters in their early (1), delayed (2) and dual time-point (RI) imaging.

See Supplementary File 2
