# Supplementary material for: Dual time-point 18F-FDG PET/CT imaging with multiple metabolic parameters in the differential diagnosis of malignancy-suspected bone/joint lesions
Source: Oncotarget. 2017 Apr 17;8(41):71188–96. doi: 10.18632/oncotarget.17140 (PMC5642630; doi:10.18632/oncotarget.17140)
Supplement: Supplementary file 2 [file oncotarget-08-71188-s002.docx]

**Supplementary Table 1: Values of different metabolic parameters in their early, delayed and dual time-point imaging.**

|  | Early | |  | Delay | |  | RI | | |
| --- | --- | --- | --- | --- | --- | --- | --- | --- | --- |
|  | Malignant | Benign | p | Malignant | Benign | p | Malignant | Benign | p |
|  | Median/25-75P | Median /25-75P |  | Median/25-75P | Median /25-75P |  | Median/25-75P | Median /25-75P |  |
| SUVmax | 6.2/4.20-8.62 | 4.22/3.15-6.39 | **0.02** | 6.94/4.63-9.96 | 5.16/3.69-8.48 | 0.05 | 0.15/0.07-0.24 | 0.11/0.06-0.28 | 0.75 |
| MTV2.0 | 45.47/21.57-109.17 | 6.16/3.42-21.32 | **0.02** | 47.62/22.52-117.67 | 7.48/1.96-24.16 | **0.01** | 0.07/0.01-0.10 | 0.03/-0.04-0.09 | 0.45 |
| Mean2.0 | 3.21/2.55-3.82 | 2.65/2.49-2.90 | 0.06 | 3.44/2.73-4.39 | 2.79/2.40-3.23 | **0.02** | 0.09/0.05-0.13 | 0.05/0.02-0.08 | **0.04** |
| TLG2.0 | 163.29/62.39-411.28 | 18.24/8.75-59.06 | 0.06 | 179.29/72.05-494.84 | 22.72/5.43-68.41 | **0.04** | 0.15/0.08-0.24 | 0.09/-0.06-0.19 | 0.57 |
| MTV2.5 | 23.67/11.59-51.87 | 2.11/0.98-10.46 | 0.06 | 33.06/13.08-62.15 | 4.75/1.17-12.62 | 0.06 | 0.10/0.04-0.35 | 0.18/0/01-0/39 | 0.28 |
| Mean2.5 | 3.68/2.98-4.26 | 3.09/2.80-3.64 | **0.02** | 4.07/3.09-4.90 | 3.24/2.91-3.70 | **0.01** | 0.09/0.05-0.13 | 0.04/0.02-0.06 | 0.50 |
| TLG2.5 | 77.62/36.63-247.41 | 7.32/3.01-34.83 | 0.12 | 143.48/44.64-286.68 | 18.13/3.73-45.05 | 0.10 | 0.23/0.09-0.47 | 0.24/-0.01-0.45 | 0.22 |
| MTV4.0 | 3.42/0.13-18.21 | 0.15/0.00-1.76 | 0.13 | 4.40/0.22-29.61 | 0.69/0.00-2.84 | 0.10 | 0.19/0.09-0.93 | 0.42/0.16-1.29 | 0.82 |
| Mean4.0 | 4.97/4.18-5.46 | 2.15/0.00-5.12 | **0.03** | 5.17/4.18-5.95 | 4.48/0.00-5.17 | 0.05 | 0.05/0.01-0.12 | 0.01/0.00-0.05 | 0.95 |
| TLG4.0 | 19.20/0.53-100.73 | 0.62/0.00-8.21 | 0.17 | 22.70/1.00-142.20 | 3.07/0.00-14.63 | 0.12 | 0.34/0.12-0.99 | 0.47/0.28-1.30 | 0.87 |
| MTV50%max | 25.23/8.93-52.12 | 11.44/3.42-29.04 | 0.10 | 20.44/9.14-39.98 | 6.66/2.64-20.83 | 0.08 | -0.02/-0.20-0.07' | -0.24/-0.34--0.044' | **0.02** |
| Mean50%max | 4.56/2.66-5.69 | 2.75/1.90-4.45 | 0.05 | 4.94/3.02-6.43 | 3.30/2.44-5.32 | 0.06 | 0.14/0.07-0.22 | 0.10/0.02-0.25 | 0.95 |
| TLG50%max | 69.01/40.34-165.93 | 39.47/9.12-74.07 | 0.12 | 76.09/48.47-163.68 | 27.25/8.70-66.23 | 0.10 | 0.10/-0.02-0.25 | -0.10/-0.24--0.024' | **0.01** |
| MTV75%max | 2.74/1.01-5.70 | 1.37/0.68-2.35 | 0.59 | 2.15/1.30-5.90 | 0.83/0.39-1.37 | 0.38 | 0/-0.25-0.53' | -0.32/-0.57--0.17' | **0.002** |
| Mean75%max | 5.45/3.59-7.17 | 3.74/2.70-5.81 | **0.04** | 6.03/3.92-8.17 | 4.43/3.29-6.97 | 0.09 | 0.15/0.07-0.22 | 0.09/0.06-0.30 | 0.55 |
| TLG75%max | 17.09/4.84-38.15 | 4.47/2.75-12.35 | 0.95 | 16.35/5.25-32.97 | 2.60/1.71-9.24 | 0.55 | 0.33/-0.10-0.76' | -0.21/-0.45--0.13' | **0.0005** |

Abbreviations SUV, standardized uptake value; Mean, SUVmean; MTV, metabolic tumor volume; TLG, total lesional glycolysis; RI, retention index; 25-75P, 25-75 percentile.
