# Supplementary material for: Dual time-point 18F-FDG PET/CT imaging with multiple metabolic parameters in the differential diagnosis of malignancy-suspected bone/joint lesions
Source: Oncotarget. 2017 Apr 17;8(41):71188–96. doi: 10.18632/oncotarget.17140 (PMC5642630; doi:10.18632/oncotarget.17140)
Supplement: Supplementary file 3 [file oncotarget-08-71188-s003.docx]

**SupplementaryTable 2: AUC, sensitivity, specificity, PLR and NLR and their** **95% confidence intervals of different metabolic parameters in their early (1), delayed (2) and dual time-point (RI) imaging.**

|  |  | **Cut-off** | **Sen/95% CI** | **Spe/95% CI** | **PLR/95% CI** | **NLR/95% CI** | **AUC/95% CI** |
| --- | --- | --- | --- | --- | --- | --- | --- |
| SUVmax | 1 | 5.07 | 68.18/45.1-86.1 | 62.86/44.9-78.5 | 1.84/1.3-2.7 | 0.51/0.2-1.1 | 0.684/0.55-0.80 |
|  | 2 | 5.69 | 63.64/40.7-82.8 | 62.86/44.9-78.5 | 1.71/1.1-2.6 | 0.58/0.3-1.2 | 0.659/0.52-0.78 |
|  | RI | 0.2748 | 31.82/13.9-54.9 | 88.57/73.3-96.8 | 2.78/1.5-5.2 | 0.77/0.3-2.0 | 0.484/0.35-0.62 |
| MTV2.0 | 1 | 16.04 | 72.73/49.8-89.3 | 88.57/73.3-96.8 | 6.36/4.8-8.4 | 0.31/0.1-1.0 | 0.818/0.69-0.91 |
|  | 2 | 8.9 | 59.09/36.4-79.3 | 94.29/80.8-99.3 | 10.34/7.2-14.8 | 0.43/0.1-1.8 | 0.795/0.67-0.89 |
|  | RI | 0.0052 | 40.91/20.7-63.6 | 80.00/63.1-91.6 | 2.05/1.2-3.5 | 0.74/0.3-1.6 | 0.574/0.44-0.70 |
| Mean2.0 | 1 | 2.74 | 68.18/45.1-86.1 | 65.71/47.8-80.9 | 1.99/1.4-2.9 | 0.48/0.2-1.0 | 0.664/0.53-0.78 |
|  | 2 | 3.37 | 81.82/59.7-94.8 | 54.29/36.6-71.2 | 1.79/1.2-2.6 | 0.33/0.1-0.9 | 0.681/0.54-0.80 |
|  | RI | 0.0794 | 81.82/59.7-94.8 | 60.00/42.1-76.1 | 2.05/1.5-2.9 | 0.30/0.1-0.8 | 0.687/0.55-0.80 |
| TLG2.0 | 1 | 41.03 | 72.73/49.8- 89.3 | 88.57/73.3-96.8 | 6.36/4.8-8.4 | 0.31/0.1-1.0 | 0.814/0.69-0.91 |
|  | 2 | 68.41 | 77.27/54.6-92.2 | 80.00/63.1-91.6 | 3.86/2.9-5.1 | 0.28/0.1-0.8 | 0.805/0.68-0.90 |
|  | RI | 0.1152 | 63.64/40.7-82.8 | 62.86/44.9-78.5 | 1.71/1.1-2.6 | 0.58/0.3-1.2 | 0.603/0.46-0.73 |
| MTV2.5 | 1 | 6.16 | 61.11/35.7-82.7 | 90.91/75.7-98.1 | 6.72/4.6-9.9 | 0.43/0.1-1.5 | 0.736/0.59-0.85 |
|  | 2 | 12.62 | 72.22/46.5-90.3 | 81.82/64.5-93.0 | 3.97/2.9-5.5 | 0.34/0.1-1.0 | 0.768/0.63-0.87 |
|  | RI | 0.1646 | 55.56/30.8-78.5 | 66.67/48.2-82.0 | 1.67/1.0-2.7 | 0.67/0.3-1.4 | 0.527/0.38-0.67 |
| Mean2.5 | 1 | 3.91 | 90.91/70.8-98.9 | 45.71/28.8-63.4 | 1.67/1.1-2.5 | 0.20/0.05-0.8 | 0.663/0.53-0.78 |
|  | 2 | 3.96 | 90.91/70.8-98.9 | 54.29/36.6-71.2 | 1.99/1.4-2.8 | 0.17/0.04-0.7 | 0.688/0.55-0.80 |
|  | RI | 0.1646 | 55.56/30.8-78.5 | 67.65/49.5-82.6 | 1.72/1.1-2.8 | 0.66/0.3-1.3 | 0.541/0.40-0.68 |
| TLG2.5 | 1 | 24.8 | 66.67/41.0-86.7 | 90.91/75.7-98.1 | 7.33/5.2-10.3 | 0.37/0.1-1.3 | 0.741/0.60-0.85 |
|  | 2 | 27.3 | 61.11/35.7-82.7 | 93.94/79.8-99.3 | 10.08/6.9-14.7 | 0.41/0.1-1.8 | 0.763/0.62-0.87 |
|  | RI | -0.0121' | 27.78/9.7-53.5 | 93.94/79.8-99.3 | 4.58/2.2-9.7 | 0.77/0.2-3.0 | 0.544/0.40-0.68 |
| MTV4.0 | 1 | 2.93 | 72.73/39.0-94.0 | 73.08/52.2-88.4 | 2.70/1.8-4.2 | 0.37/0.1-1.2 | 0.663/0.49-0.81 |
|  | 2 | 5.38 | 72.73/39.0-94.0 | 65.38/44.3-82.8 | 2.10/1.3-3.3 | 0.42/0.1-1.3 | 0.671/0.50-0.82 |
|  | RI | 0.2266 | 72.73/39.0-94.0 | 61.54/40.6-79.8 | 1.89/1.2-3.0 | 0.44/0.2-1.3 | 0.579/0.42-0.74 |
| Mean4.0 | 1 | 4.45 | 68.18/45.1-86.1 | 62.82/44.9-78.5 | 1.84/1.3-2.7 | 0.51/0.2-1.1 | 0.660/0.52-0.78 |
|  | 2 | 5.39 | 90.91/70.8-98.9 | 45.71/28.8-63.4 | 1.67/1.1-2.5 | 0.20/0.05-0.8 | 0.656/0.52-0.78 |
|  | RI | 0.0365 | 72.73/39.0-94.0 | 62.96/42.4-80.6 | 1.96/1.2-3.1 | 0.43/0.1-1.3 | 0.643/0.47-0.79 |
| TLG4.0 | 1 | 13.89 | 72.73/39.0-94.0 | 73.08/52.2-88.4 | 2.7/1.8-4.2 | 0.37/0.1-1.2 | 0.657/0.48-0.81 |
|  | 2 | 18.83 | 63.64/30.8-89.1 | 76.92/56.4-91.0 | 2.76/1.7-4.5 | 0.47/0.2-1.4 | 0.661/0.49-0.81 |
|  | RI | 0.3863 | 63.64/30.8-89.1 | 61.54/40.6-79.8 | 1.65/1.0-2.8 | 0.59/0.2-1.5 | 0.556/0.38-0.72 |
| MTV50%max | 1 | 7.53 | 50.00/28.2-71.8 | 82.86/66.4-93.4 | 2.92/1.9-4.5 | 0.6/0.3-1.4 | 0.659/0.52-0.78 |
|  | 2 | 6.67 | 54.55/32.2-75.6 | 88.57/73.3-96.8 | 4.77/3.2-7.1 | 0.51/0.2-1.4 | 0.712/0.58-0.82 |
|  | RI | -0.1215 | 68.18/45.1-86.1 | 68.57/50.7-83.1 | 2.17/1.5-3.1 | 0.46/0.2-1.0 | 0.686/0.55-0.80 |
| Mean50%max | 1 | 4.72 | 86.36/65.1-97.1 | 48.57/31.4-66.0 | 1.68/1.1-2.5 | 0.28/0.09-0.8 | 0.686/0.55-0.80 |
|  | 2 | 5.92 | 90.91/70.8-98.9 | 40.00/23.9-57.9 | 1.52/1.0-2.3 | 0.23/0.06-0.9 | 0.662/0.53-0.78 |
|  | RI | 0.1165 | 59.09/36.4-79.3 | 65.71/47.8-80.9 | 1.72/1.1-2.6 | 0.62/0.3-1.2 | 0.562/0.43-0.69 |
| TLG50%max | 1 | 14.88 | 40.91/20.7-63.6 | 100.0/90.0-100.0 | -/- | -/- | 0.716/0.58-0.83 |
|  | 2 | 39.02 | 68.18/45.1-86.1 | 82.86/66.4-93.4 | 3.98/2.9-5.5 | 0.38/0.1-1.0 | 0.781/0.65-0.88 |
|  | RI | -0.024 | 77.27/54.6-92.2 | 74.29/56.7-87.5 | 3.01/2.2-4.1 | 0.31/0.1-0.8 | 0.732/0.60-0.84 |
| MTV75%max | 1 | 2.35 | 77.27/54.6-92.2 | 57.14/39.4-73.7 | 1.80/1.3-2.6 | 0.40/0.2-0.9 | 0.630/0.49-0.75 |
|  | 2 | 0.98 | 68.18/45.1-86.1 | 82.86/66.4-93.4 | 3.98/2.9-5.5 | 0.38/0.1-1.0 | 0.790/0.66-0.89 |
|  | RI | 0.1136 | 100/84.6-100.0 | 48.57/31.4-66.0 | 1.94/1.4-2.7 | 0/- | 0.769/0.64-0.87 |
| Mean75%max | 1 | 4.11 | 63.64/40.7-82.8 | 68.57/50.7-83.1 | 2.02/1.4-3.0 | 0.53/0.3-1.1 | 0.675/0.54-0.79 |
|  | 2 | 5.81 | 72.73/49.8 - 89.3 | 54.29/36.6 - 71.2 | 1.59/1.1 - 2.4 | 0.50/0.2 - 1.1 | 0.643/0.51-0.77 |
|  | RI | 0.1152 | 63.64/40.7-82.8 | 65.71/47.8-80.9 | 1.86/1.2-2.8 | 0.55/0.3-1.1 | 0.531/0.39-0.67 |
| TLG75%max | 1 | 5.661 | 68.18/45.1-86.1 | 74.29/56.7-87.5 | 2.65/1.9-3.7 | 0.43/0.2-1.0 | 0.705/0.57-0.82 |
|  | 2 | 3.401 | 68.18/45.1-86.1 | 88.57/73.3-96.8 | 5.97/4.4-8.1 | 0.36/0.1-1.1 | 0.819/0.70-0.91 |
|  | RI | 0.1109 | 95.45/77.2-99.9 | 60.00/42.1-76.1 | 2.39/1.8-3.2 | 0.076/0.01-0.5 | 0.794/0.67-0.89 |

Abbreviations SUV, standardized uptake value; Mean, SUVmean; MTV, metabolic tumor volume; TLG, total lesional glycolysis; RI, retention index; Sen, sensitivity;Spe, specificity; PLR, positive likelihood ratio; NLR, negative likelihood radio; AUC, areas under the curve; CI, confidence interval.
